# CPW Fed Compact UWB 4-Element MIMO Antenna with High Isolation

**DOI:** 10.3390/s21082688

**Published:** 2021-04-11

**Authors:** Wenfei Yin, Shaoxiang Chen, Junjie Chang, Chunhua Li, Salam K. Khamas

**Affiliations:** 1 School of Computer Science and Information Engineering, Hefei University of Technology, Hefei 230009, China; shaoxiangc2020@sina.com (S.C.); junjiec2021@sina.com (J.C.); lch2014@hfut.edu.cn (C.L.); 2 Department of Electronic and Electrical Engineering, University of Sheffield, Sheffield S10 2TN, UK; s.khamas@sheffield.ac.uk

**Keywords:** UWB, MIMO antenna, coplanar waveguide (CPW), high isolation, stub

## Abstract

In the paper, an extremely compact multiple-input-multiple-output (MIMO) antenna is proposed for portable wireless ultrawideband (UWB) applications. The proposed prototype consists of four monopole antenna elements, which are placed perpendicularly to achieve polarization diversity. In addition, the mutual coupling between antenna elements is suppressed by designing the gap between the radiation element and the ground plane. Moreover, a matching stub has been connected to the feedline to ensure impedance matching in high frequency. Both simulated and measured results indicate that the proposed antenna has a bandwidth of 3–20 GHz, with a high isolation better than 17 dB. In addition, the designed MIMO antenna offers excellent radiation characteristics and stable gain over the whole working band. The envelope correlation coefficient (ECC) is less than 0.1, which shows that the antenna can meet the polarization diversity characteristics well.

## 1. Introduction

Federal Communications Commission (FCC) has developed a frequency range (3.1–10.6 GHz) for commercial applications but limits the power transmission to low levels [1]. This could result in a severe multipath signal fading owing to substantial surrounding’s scattering and deterioration of the transmission overall performance of the ultrawideband (UWB) devices [2]. Multiple-input-multiple-output (MIMO) technology has been applied successfully to improve the channel capacity and link quality by producing multiplexing gain as well as diversity gain, respectively [3]. Additionally, the main challenge faced by many researchers is the design of UWB-MIMO antennas with high isolation when placed within a compact portable device size [4]. The antenna miniaturization affects the working bandwidth and radiation efficiency to a great extent. Therefore, when numerous antennas are integrated into a rather small terminal, a strong mutual coupling between elements is inevitable, which leads to antenna impedance mismatching, pattern deterioration and lower channel capacity.

In recent years, the design of a compact high isolation MIMO antenna has become a major issue. Many scholars have studied different methods to improve the isolation of MIMO antennas. One of the most effective approaches is to place the feedlines perpendicularly to each other, which results in considerable polarization and pattern diversity [5,6,7]. In addition, various defected ground plane structures incorporating slots and stubs have been used to improve the isolation of MIMO antennas [8,9,10,11,12]. Furthermore, novel miniaturized two-layer electromagnetic band gap (EBG) structures have been presented to minimize the electromagnetic coupling between closely spaced UWB planar monopoles on a common ground plane [13]. In addition, a wideband neutralization line has been proposed to reduce the mutual coupling of a compact UWB-MIMO antenna [14].

Recently, MIMO designs that support wider working frequency bands have received considerable research interests [15,16,17]. For example, a compact 4-element MIMO antenna has been proposed for portable wireless UWB applications with a wide frequency band that extends from 3.1 to 12 GHz [15]. In addition, a highly isolated compact 4-element planar UWB-MIMO antenna that operates over a bandwidth of 3–15 GHz has been reported [16]. In a recent study, a novel UWB-MIMO antenna system with high isolation has been proposed with a frequency range of 2.9 to 40 GHz and a mutual coupling that is less than −17 dB [17]. Moreover, it can also be observed from the recent literature that 4-element antennas have become the mainstream of UWB-MIMO antenna research. However, a compact 4-element UWB-MIMO antenna with UWB (3.1–20 GHz) has not been reported earlier in the open literature.

In this study, a compact UWB 4-element MIMO antenna with high isolation is proposed. The proposed antenna has sufficient channel capacity and can be used for precision positioning applications. To the best of authors’ knowledge, the novelty of this work is the size of whole antenna is extremely compacted designed in the working frequency band extends from 3.1–20 GHz. Furthermore, the 4 antenna elements allow for a significant increase in channel capacity compared to the 2 elements design. In addition, a stub is specially designed to ensure the impedance matching during the working frequency. Furthermore, the proposed antenna offers a radiation efficiency of more than 75%. The achieved envelope correlation coefficient (ECC) demonstrates that the antenna satisfies the polarization diversity characteristic requirements. These appealing characteristics have been achieved with an overall antenna size of 38 × 38 × 1.6 mm^3^. The simulations have been conducted using the High Frequency Structure Simulator (HFSS). A prototype has been built and measured with close agreement between experimental and simulated results.

## 2. Antenna Design and Structure

### 2.1. CPW Feed

A coplanar waveguide (CPW)-fed microstrip patch antenna structure has been considered using an FR4 substrate with dielectric constant of *ε_r_* = 4.4, loss tangent of 0.02 and thickness of 1.6 mm. The effective permittivity of the coplanar waveguide configuration is given by [18]:

(1) $$\varepsilon_{nc}=\frac{\varepsilon_{r}+1}{2}\{\tanh[0.775\ln(h/G)+1.75]+\frac{kG}{h}\left.\times \left[0.004-0.7\;k+0.01\left(1-0.1\varepsilon_{r}\right)(0.25+k)\right]\right\}$$

where

(2) $$k=\frac{W}{W+2G}.$$

*W* is the width of the central conduction band, *G* is the gap between the conduction band and the ground and *h* is the thickness of the dielectric substrate, the characteristic impedance of the CPW line can be expressed by the elliptic function *K (k)* of the first kind [18]:

(3) $$Z_{0_{CPW}}=\frac{30\pi }{{\sqrt{\varepsilon }}_{re}}\frac{K^{\prime }(k)}{K(k)}.$$

in which

(4) $$\frac{K^{\prime }(k)}{K(k)}=\left[\frac{\pi }{\ln\left(2\frac{1+\sqrt{k^{\prime }}}{1-\sqrt{k^{\prime }}}\right)}\right]\;\;\mathrm{if}\;0<k<0.707=\left[\frac{\ln\left(2\frac{1+\sqrt{k^{’}}}{1-\sqrt{k^{’}}}\right)}{\pi }\right]\;\;\mathrm{if}\;0.707<k<1.$$

where, $k^{’}=\sqrt{1-k^{2}}$,$K^{\prime }(k)=K(k^{\prime })$.

The width of the central guide band, gap between the guide band and the ground have been chosen as 1.5 mm and 0.5 mm, respectively. Substituting the relevant parameters into Equation (3), the characteristics impedance of the CPW feeder can be calculated as $Z_{0_{CPW}}\approx 51\;\Omega$.

### 2.2. Antenna Configuration

#### 2.2.1. Single Element

**Figure 1:**
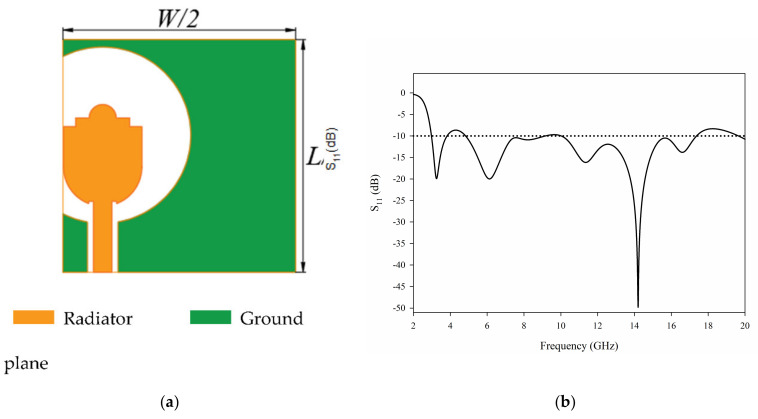
The initial single element antenna: (**a**) geometry (**b**) S_11_.

The configuration of the proposed UWB MIMO antenna is showed as Figure 1. At first, we consider a single element, the initial model is described as Figure 1a. The antenna structure is simulated in HFSS, and the S parameters of the antenna are obtained, as shown in Figure 1b.

**Figure 2:**
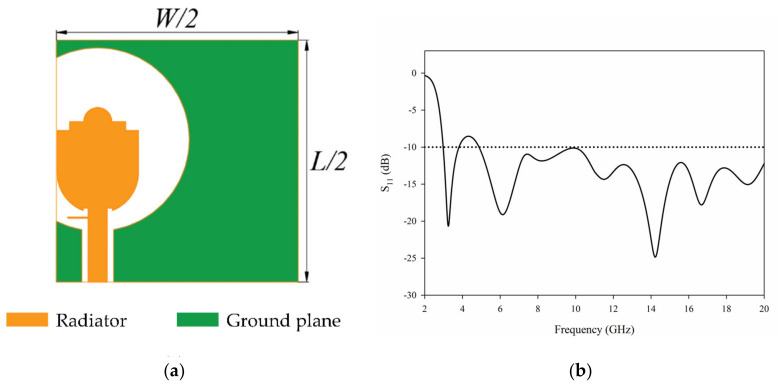
The single element antenna with stub: (**a**) geometry (**b**) S_11_.

It can be seen from the S parameter that the single element antenna has the possibility of working in UWB. However, the performance of the antenna deteriorates after 17 GHz. In order to improve the performance of the antenna further, we add parasitic branches to the feeder. The antenna structure with stub is shown in Figure 2a, and the antenna S parameters are shown in Figure 2b.

Obviously, after adding the stub, the matching of single element antenna in the frequency band high than 10.6 GHz has been greatly improved. Next, we can further design the 4-element MIMO antenna.

#### 2.2.2. MIMO Antenna

**Figure 3:**
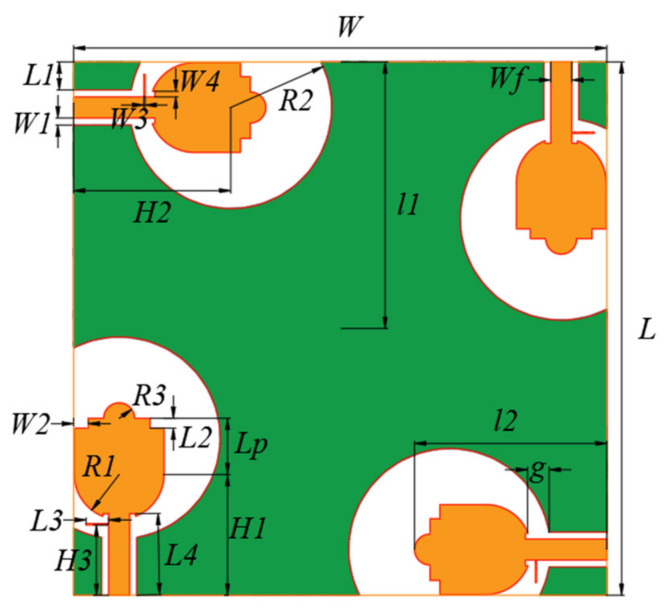
Geometry of the proposed multiple-input-multiple-output (MIMO) antenna.

The final geometry of the proposed UWB-MIMO antenna is illustrated in Figure 3 and the design parameters are listed in Table 1. The antenna structure and size have been selected to meet the operating frequency requirements. The first resonant frequency of the proposed monopole antenna can be approximately calculated as [19]:

(5) $$f_{r}=\frac{144}{l_{1}+l_{2}+g+\frac{A_{1}}{2\pi l_{1}\sqrt{\varepsilon_{re}}}+\frac{A_{2}}{2\pi l_{2}\sqrt{\varepsilon_{re}}}}.$$

In Equation (5), *A*_1_ and *A*_2_ represent the areas of the ground plane and radiating patch, respectively, *l*_1_ and *l*_2_ represent the length of the ground plane and radiating patch respectively and g represents the distance between the ground and the radiation patch. All parameters are in the units of mm. For the considered antenna, *l*_1_ = 19 mm, *l*_2_ = 13.7 mm, *g* = 1.56 mm, *A*_1_ = 42.2 mm^2^ and *A*_2_ = 224.06 mm^2^. The calculated and simulated *fr* have been obtained as 4 and 3.7 GHz, respectively. It can be seen that the estimated value is close to the simulated value.

The radiating patch and ground plane have been printed on the surface of the substrate. The used patch shape is evolved from a circular monopole. The size of the half around ground structure greatly affects the impedance matching of the antenna. The semicircular protrusion structure, with a radius of *R*_3_, at the top of the patch and the stub connected to the feeder line can both improve the antenna matching to achieve the ultrawideband operation. In particular, the stub that plays an important role in the impedance matching. In addition, the small rectangular notches on the left and right-hand sides of the semicircular protrusion are denoted as *W*_2_ and *L*_2_ and they help with the impedance matching.

## 3. Influence of Special Structure

### 3.1. Semi Surround Ground Structure

**Figure 4:**
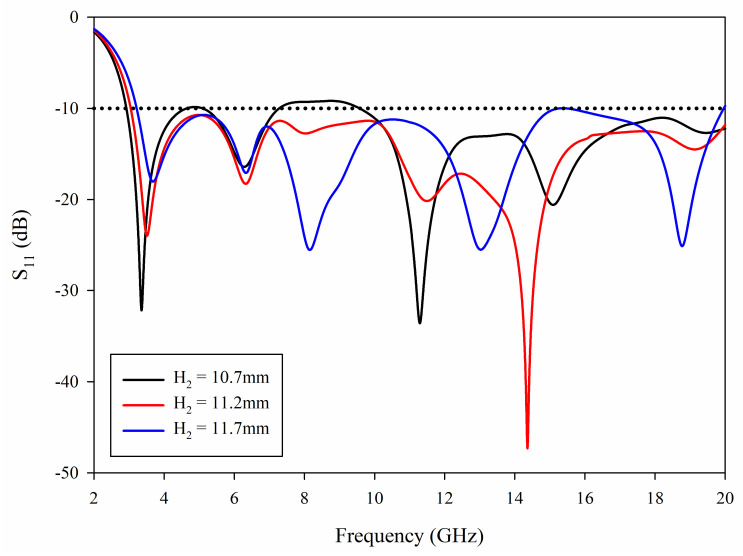
Simulated S_11_ with different *H*_2_.

The half around ground structure has a great influence on the impedance matching bandwidth over the whole frequency band, especially its position *H*_2_. The effects of different values of *H*_2_ are studied and the results are shown in Figure 4.

It can be noted from Figure 4 that that by increasing *H*_2_, the first resonant frequency point shifts from 3.2 to 3.8 GHz. This is because when *H*_2_ is increased, the gap between the radiating patch and the surrounding ground plane is reduced, that is to say, *g* in Equation (5) is decreased, resulting in the shift of the first resonant frequency point. In addition, it can be observed that when *H*_2_ is shorter, a narrower bandwidth is achieved since the match is lost around 5 GHz and 8–9 GHz. When *H*_2_ is longer, the cutoff frequency is about 3.2 GHz, and the matching is lost around 15 GHz, which fails to meet the requirements. Therefore, by adjusting *H*_2_ to a suitable value, the matching can be achieved in the range of 3–20 GHz.

### 3.2. Length of Radiating Patch

**Figure 5:**
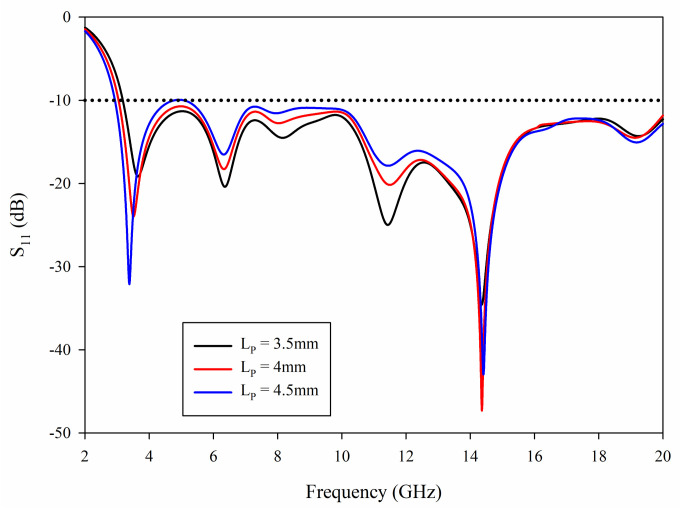
Simulated S_11_ with different *Lp*.

The length of the radiating patch, *Lp*, has slight effect on the antenna matching beyond 10.6 GHz, but has a greater influence on the ultrawideband. Figure 5 presents the variations of S_11_ for various patch lengths.

From Figure 5, it can be noted that with the increase of *Lp*, the first resonant frequency point moves to the left. This is because when *Lp* increases, the length of radiating patch will increase, that is, *l*_1_ in Equation (5) will increase, which will cause the first resonant frequency shift to the lower frequency, meanwhile, the cut-off frequency of low frequency will also shift to the lower frequency. When *Lp* is small, the cut-off frequency is approximately 3.2 GHz, which fails to satisfy the requirements of UWB. In addition, with the increase of *Lp*, the antenna matching in the 4–6 and 7–10 GHz bands becomes worse. Selecting appropriate *Lp* can make the antenna achieve better impedance matching in the UWB operating frequency range.

### 3.3. A Stub Connected to Feedline

**Figure 6:**
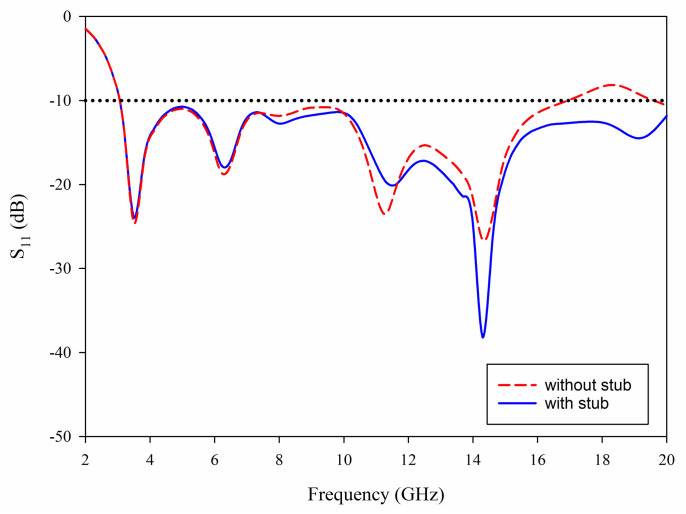
Simulated S_11_ without stub and with stub.

The stub connected to feedline plays an important role in the improvement of matching in the 10.6–20 GHz band. Figure 6 illustrates the comparison of S_11_ with and without stub, where it can be observed that a sufficiently wide matching bandwidth has been achieved up to 17 GHz. Moreover, this can be extended by adding the matching stub that has created another resonance point around 15 GHz, which effectively extends the impedance matching to cover the whole frequency range. 

## 4. Results and Discussions

### 4.1. S-Parameters

**Figure 7:**
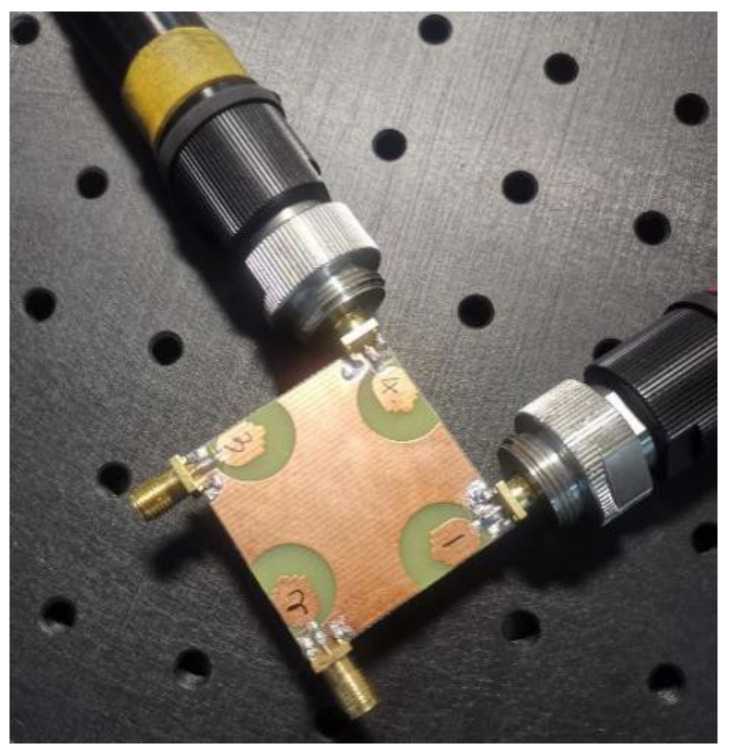
Fabrication photograph of proposed coplanar waveguide (CPW) fed compact ultrawideband (UWB)-MIMO antenna.

**Figure 8:**
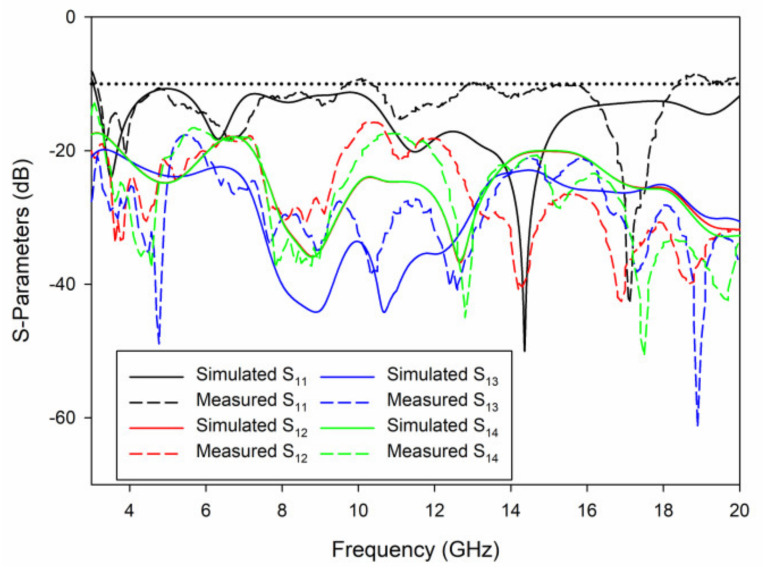
Measured and simulated S-parameters for the proposed UWB-MIMO antenna.

As can be seen in Figure 7, the proposed antenna in this paper has been fabricated, and S-parameter measurement has been tested by using Rohde & Schwarz (Munich, Germany) ZVA 40. It can be seen from Figure 8 that the measurement results are basically consistent with the simulation results, and antenna ports show very good impedance matching bandwidth from 3 to 20 GHz. The isolation between the four ports is better than 17 dB in the whole bandwidth. However, it should be noted that the measured S_11_ of the proposed UWB-MIMO antenna system are not totally identical with the simulated results at high frequencies. This could be attributed to fabrication and experimental tolerances with respect to the antenna printing, welding as well as testing conditions.

### 4.2. Surface Current Distribution

**Figure 9:**
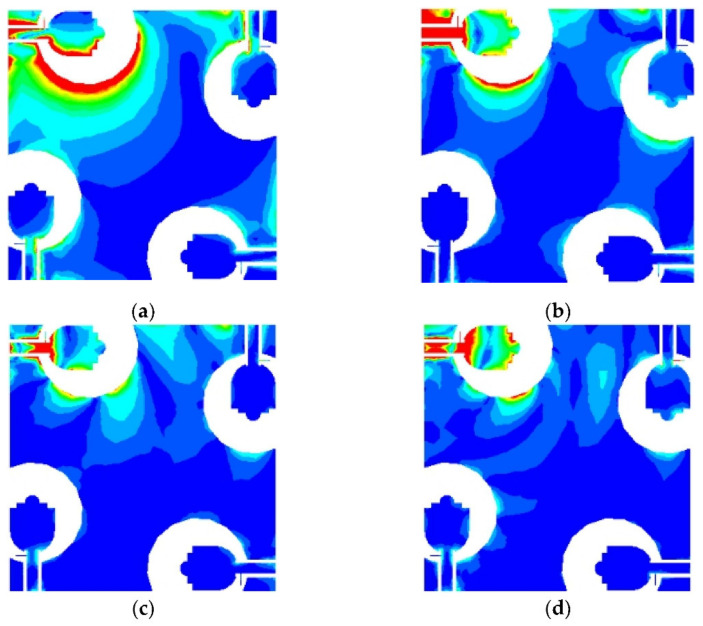
Surface current distributions of the MIMO antenna at: (**a**) 3.5 GHz, (**b**) 9 GHz, (**c**) 14 GHz, (**d**) 19 GHz.

In order to achieve the antenna easy fabrication and meet the requirements of high isolation between antenna ports. Firstly, the four antenna elements are placed perpendicularly to achieve polarization diversity. In addition, the mutual coupling between antenna elements is suppressed by designing the gap between the radiation element and the ground plane. Figure 9 describes the surface current distribution of the antenna at 3.5 GHz, 9 GHz, 14 GHz and 19 GHz. When port 1 is excited, each one of the remaining ports is terminated with a 50 Ω load. It can be seen from the figure that the current is concentrated around the source-fed element, limited coupling currents flow into other elements. These results confirm the achieved good isolation performance of the proposed design through the whole frequency band. Moreover, a high current density for the stub at 19 GHz, which also reflects that the introduction of stub has considerably improved the matching of the antenna at high frequency.

### 4.3. Radiation Patterns and Gain

**Figure 10:**
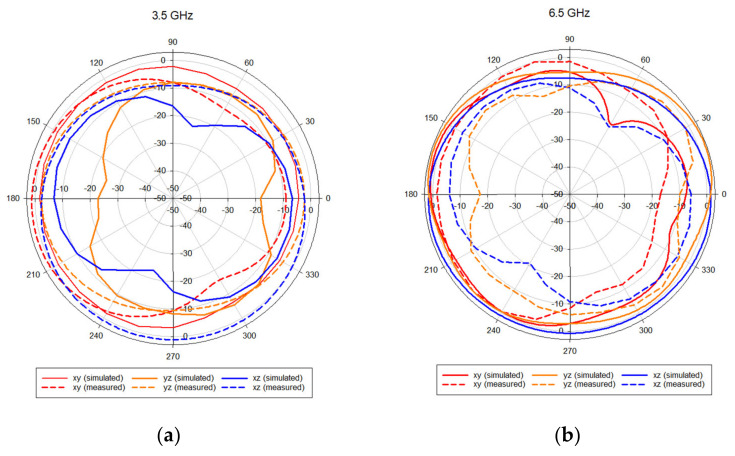

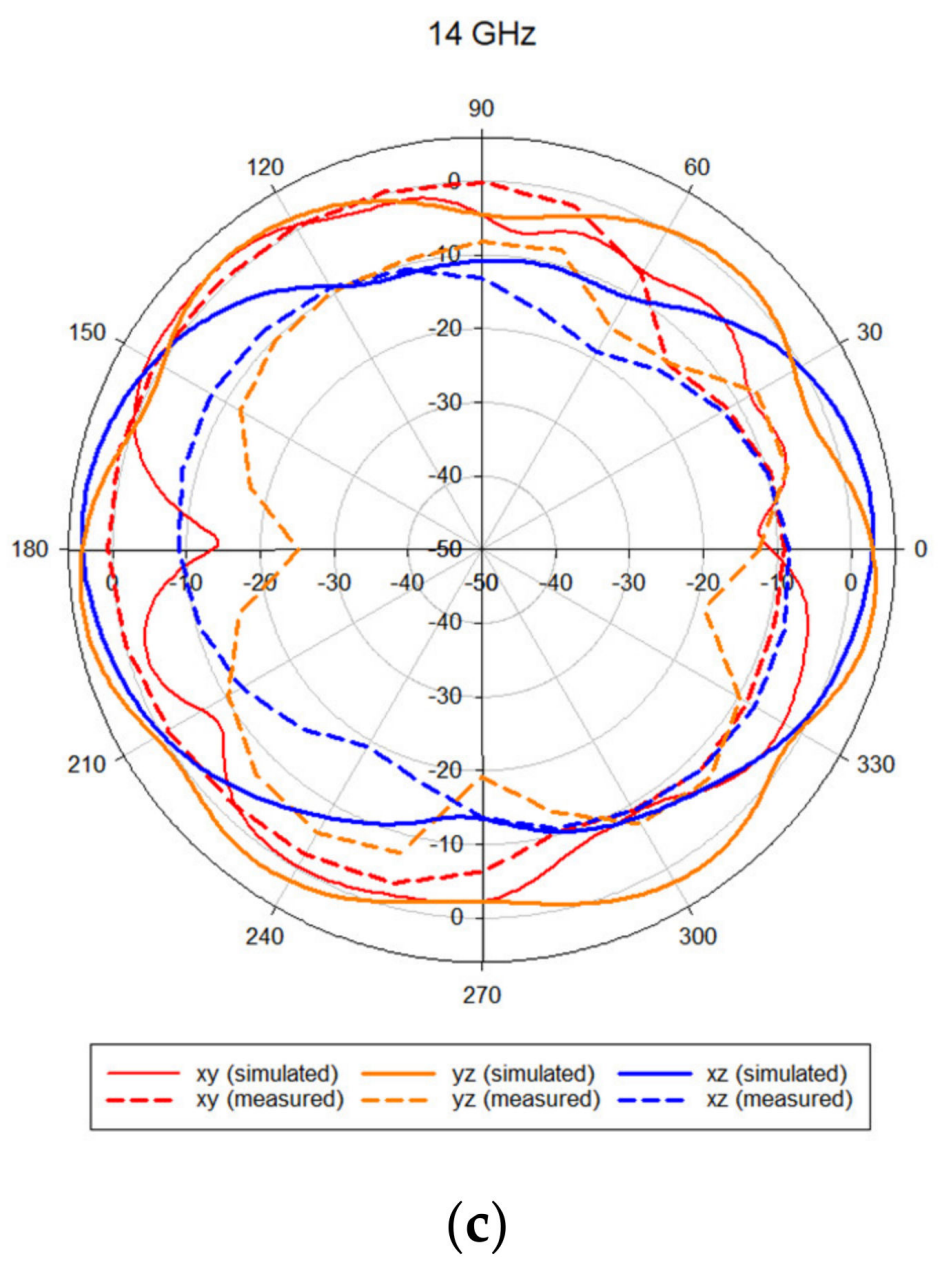
Radiation pattern for the proposed MIMO antenna at: (**a**) 3.5 GHz, (**b**) 6.5 GHz, (**c**) 14 GHz.

**Figure 11:**
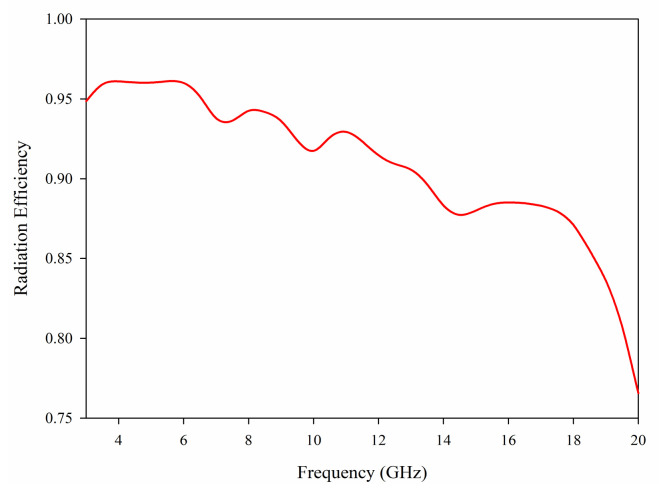
Radiation efficiency of the proposed MIMO antenna.

The far field radiation patterns of the proposed antenna are presented in Figure 10 at the four operating frequencies of 3.5 GHz, 6.5 GHz and 14 GHz. The patterns demonstrate good omnidirectional characteristics on the *xy*, *xz* and *yz* planes, which means the antenna receives electromagnetic waves from all directions. The results also confirm that stable radiation characteristics have been maintained at various frequencies. Figure 11 presents the radiation efficiency in the case of port 1 excitation, where it can be observed that the typical efficiency of the proposed MIMO antenna is above 75% over the entire frequency band of 3–20 GHz.

### 4.4. MIMO Performance

As an index to evaluate the correlation of MIMO antenna radiation patterns, Envelope correlation coefficient (ECC) can usually be calculated by far field radiation parameters. What is more, the ECC is required to be less than 0.5 to ensure the antenna performance. The ECC is calculated according to Equation (6) [20]:

(6) $$\rho_{eij}=\frac{{\left|\int_{0}^{2\pi }\int_{0}^{\pi }\left(XPR\cdot E_{\theta i}\cdot E_{\theta j}^{*}\cdot P_{\theta }+E_{\varphi i}\cdot E_{\varphi j}^{*}\cdot P\varphi \right)d\Omega \right|}^{2}}{\int_{0}^{2\pi }\int_{0}^{\pi }\left(XPR\cdot E_{\theta i}\cdot E_{\theta i}^{*}\cdot P_{\theta }+E_{\varphi i}\cdot E_{\varphi i}^{*}\cdot P\varphi \right)d\Omega \times \int_{0}^{2\pi }\int_{0}^{\pi }\left(XPR\cdot E_{\theta j}\cdot E_{\theta j}^{*}\cdot P_{\theta }+E_{\varphi j}\cdot E_{\varphi j}^{*}\cdot P\varphi \right)d\Omega }$$

The envelope correlation coefficient (ECC) *ρ_eij_* is calculated when *N* = 4 throughout the whole bandwidth. Diversity gain (DG) can also be used to express the correlation of antennas, which can be calculated by Equation (7) [21]. The smaller the ECC value, the larger the diversity gain of the antenna.

(7) $$\mathrm{DG}=10\sqrt{1-{\mathrm{ECC}}^{2}}.$$

**Figure 12:**
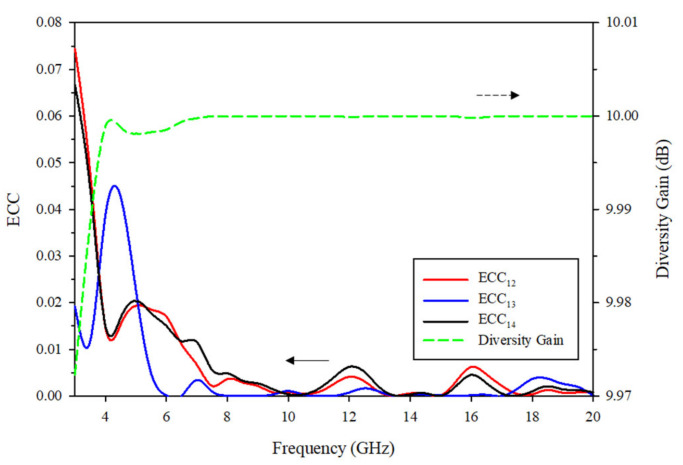
ECC and diversity gain of the proposed MIMO antenna.

The ECC and DG of the proposed UWB-MIMO antenna system are plotted in Figure 12, where it can be observed that for all the elements, ECC is rather less than 0.08 over the entire impedance bandwidth and is less than 0.02 from 6 to 20 GHz, while the DG is greater than 9.97. These results reveal that the designed antenna fulfill the requirement of achieving smaller ECC and larger DG at the same time.

The signal-to-noise ratio between imperfect MIMO antenna and the ideal antenna is defined as multiplexing efficiency (*η_mux_*) and written by Equation (8) [22]:

(8) $$\eta_{mux}=\sqrt{\eta_{i}\eta_{j}(1-{\left|\rho_{c}\right|}^{2})}.$$

**Figure 13:**
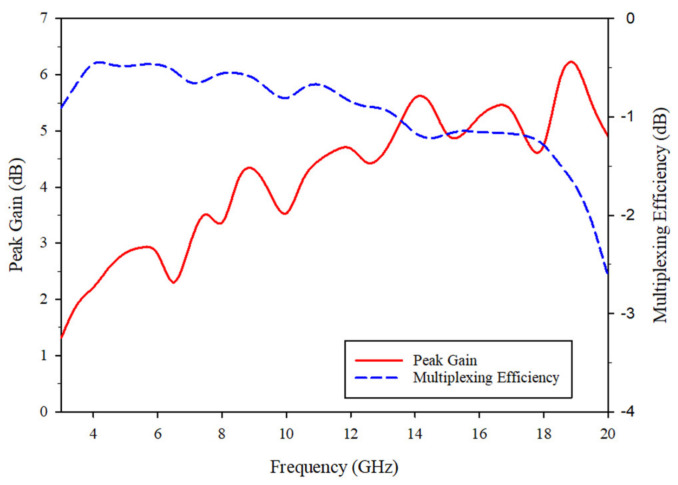
Peak Gain and multiplexing efficiency of the proposed MIMO antenna.

As demonstrated in Figure 13, *η_mux_* is higher than −3 dB throughout the whole frequency range. In addition, the peak gain is more than 1.3 dBi across the entire bandwidth. Therefore, both parameters satisfy the requirements of MIMO wireless communication systems.

### 4.5. Performance Comparison

In order to highlight the advantages of proposed antennas, the performances of antennas in different literatures are summarized in Table 2. Compared with [6,9,14], the proposed antenna has a wider bandwidth. Secondly, the proposed antenna has more ports than [6,9,11,12,15]. In terms of antenna compactness, the proposed antenna saves the most space than [17,18] in the case of the same number of ports.

## 5. Conclusions

A novel 4-element UWB-MIMO CPW-fed antenna has been proposed with a compact size of only 38 × 38 × 1.6 mm^3^. The antenna achieves a considerably wide impedance bandwidth from 3–20 GHz. Easy fabrication decoupling structure is utilized to design the proposed antenna. The isolation between various elements is less than −17 dB. The incorporation of a matching stub is placed on the feeder of the antenna to improve the impedance matching in the high frequency band. All the simulated and measured results demonstrate that the proposed antenna offers important characteristics such as ultra-wide bandwidth, low mutual coupling, stable gain and radiation patterns. In addition, low ECC demonstrates the potential of the proposed antenna with presented diversity characteristics. A comparison of the proposed MIMO antenna with the other reported antenna structures has been presented to highlight the novelty and significance of our proposed work. Therefore, the CPW Fed Compact UWB 4-Element MIMO Antenna can be considered as a promising candidate for UWB applications. 

## Figures and Tables

**Table 1:** Antenna dimensions shown in Figure 1 (unit: mm).

*W*	*Wf*	*W*_1_	*W*_2_	*W*_3_	*W*_4_	*H*	*H*_1_	*H*_2_	*H*_3_	*g*
38	1.5	0.5	1	0.1	0.4	1.6	8.6	11.2	5	1.56
*L*	*L*p	*L*_1_	*L*_2_	*L*_3_	*L*_4_	*R*_1_	*R*_2_	*R*_3_	*l*_1_	*l*_2_
38	4	2	0.7	1.6	5.8	3.2	7.2	1.1	19	13.7

**Table 2:** Performance comparisons with the recently published designs.

Reference	Antenna Size (mm^2^)	Bandwidth (GHz)	Gain (dBi)	Isolation (dB)	ECC	Ports
[6]	35 × 35	3.0–12	NA	>20	<0.3	2
[9]	30 × 40	3.1–10.6	NA	>15	<0.15	2
[11]	34 × 18	2.93–20	0–7	>22	<0.01	2
[12]	50 × 30	2.5–14.5	0.1–4	>20	<0.04	2
[15]	36 × 18	3.2–12	0–7	>22	<0.01	2
[17]	58 × 58	2.9–40	4.3–13.5	>17	<0.01	4
[18]	80 × 80	2.1–20	5.8 average	>25	<0.02	4
This work	38 × 38	3.0–20	1.3–6.2	>17	<0.08	4

## Data Availability

Not applicable.
